# Vacuolated Marrow Cytopenias from Copper Deficiency to *UBA1*-Mutant VEXAS: Molecular Landscape, Systematic Review, and Cost-Efficient Diagnostic Algorithm

**DOI:** 10.3390/ijms26168044

**Published:** 2025-08-20

**Authors:** Akiyoshi Takami, Kaori Uchino, Mai Terashima, Seiko Sato, Yuka Kondo, J. Luis Espinoza, Megumi Enomoto

**Affiliations:** 1 Division of Hematology, Department of Internal Medicine, Aichi Medical University School of Medicine, Nagakute 480-1195, Japan; ksakai@aichi-med-u.ac.jp; 2 Department of Central Clinical Laboratory, Aichi Medical University Hospital, Nagakute 480-1195, Japan; terashima.mai.092@mail.aichi-med-u.ac.jp (M.T.); menomo@aichi-med-u.ac.jp (M.E.); 3 Department of Clinical Laboratory, Fujita Health University Hospital, Toyoake 470-1192, Japan; s-mano@fujita-hu.ac.jp; 4 Department of Central Clinical Laboratory, Toyohashi Municipal Hospital, Toyohashi 441-8570, Japan; kondo-yuka@toyohashi-mh.jp; 5 Faculty of Health Sciences, Kanazawa University, Kanazawa 920-0942, Japan; luis@staff.kanazawa-u.ac.jp

**Keywords:** vacuoles, cytopenia, copper deficiency, VEXAS, *UBA1*, systematic review, diagnostic algorithm

## Abstract

Although cytoplasmic vacuoles are instantly recognizable on bone marrow smears, their etiologic significance remains a diagnostic dilemma in everyday practice. We conducted a PRISMA (Preferred Reporting Items for Systematic reviews and Meta-Analyses) aligned systematic review with narrative synthesis registered in PROSPERO and refrained from meta-analysis owing to high between-study heterogeneity (I^2^ > 80%). Across 22 studies, we identified 818 unique adults with vacuolated marrow cytopenias. A stepwise diagnostic algorithm—serum copper/ceruloplasmin followed, when non-diagnostic, by *UBA1* hotspot sequencing in adults meeting clinical “red flags”—correctly classified 97% of cases within <5 days at a median laboratory cost of ~USD 173, reserving broad myeloid next-generation sequencing for the atypical minority. This synthesis clarifies the relative frequency of major etiologies and provides a cost-efficient, practice-ready pathway that bridges trace-metal metabolism and somatic genomics.

## 1. Introduction

Although cytoplasmic vacuoles are instantly recognizable under the microscope, their etiologic significance remains a diagnostic dilemma in everyday practice. A recent state-of-the-art review [1] stresses that the classic literature links large, lipid-poor vacuoles to copper deficiency, alcohol misuse, or drug toxicity, yet these causes explain only a fraction of post-2020 cases [2,3]. The 2020 discovery of somatic *UBA1*-mutant VEXAS (vacuoles, E1 enzyme, X-linked, autoinflammatory, somatic) syndrome further reshaped the differential: this late-onset autoinflammatory disease features prominent vacuoles and often presents with otherwise unexplained cytopenia [4]. More than 700 patients have since been reported, but data remain fragmented across small series, impeding reliable prevalence estimates and rational test ordering.

Clinicians thus face a practical dilemma: should they begin with serum copper studies, order targeted *UBA1* sequencing, proceed directly to broad myeloid next-generation sequencing (NGS), or pursue cytogenetics when confronted with vacuolated cytopenia? Without an evidence-weighted pathway, reversible etiologies such as copper deficiency may be overlooked, whereas costly or invasive investigations may be overused.

Here, we consolidate current knowledge by two complementary approaches. First, we performed a PRISMA (Preferred Reporting Items for Systematic reviews and Meta-Analyses) aligned systematic review to quantify the relative frequencies, clinical profiles, and morphologic hallmarks of vacuolated marrow cytopenias in adults. Second, guided by the evolving literature—including the insights of the recent review by Elbadry and Mabed [1]—we integrate these data with post-2020 pathobiology to propose a pragmatic four-step diagnostic algorithm that balances diagnostic yield, turnaround time, and cost. This narrative synthesis aims to guide daily hematology practice while highlighting areas where prospective studies are urgently needed.

## 2. Methods

### 2.1. Framework and Registration

We embedded a systematic review within a narrative review, following the PRISMA 2020 statement [5]. The protocol was registered prospectively in PROSPERO (CRD420251082738) [6], and the full search strings, checklist, and protocol are reproduced in Supplementary Protocol S1.

We performed a PRISMA-aligned systematic review with narrative synthesis, registered in PROSPERO, and prespecified that no quantitative meta-analysis would be undertaken given substantial between-study heterogeneity (I^2^ > 80%). Wherever available, we extracted individual-level data and summarized simple proportions with exact binomial confidence intervals.

### 2.2. Eligibility Criteria

We considered reports that analyzed adults (≥18 years) with single-lineage or multilineage cytopenia in whom cytoplasmic vacuoles were demonstrated in at least one hematopoietic precursor compartment on light microscopy. Acceptable study designs were cohort studies, cross-sectional analyses, or case series that enrolled three or more patients and provided primary diagnostic or outcome data. Single-case reports, narrative reviews, conference abstracts without full texts, pediatric studies, and non-English publications were excluded. We queried PubMed, Web of Science Core Collection, and CENTRAL on 31 May 2025 using a search window that began 31 December 2020, the day prior to the first description of VEXAS. To capture the earlier literature on copper-deficiency cytopenia, we performed backward citation-chasing and keyword hand-searching (2000–2020), which yielded three additional cohort reports not indexed by the electronic filters.

### 2.3. Information Sources and Search Strategy

With support from a medical librarian, we searched PubMed, Web of Science Core Collection, and Cochrane Central Register of Controlled Trials (CENTRAL) on 31 May 2025 (Supplementary Table S1). The core PubMed string was:

(“vacuole”[Title/Abstract] OR “vacuolated”[Title/Abstract]) AND (“bone marrow”[Title/Abstract] OR “marrow”[Title/Abstract]) AND (cytopenia OR anemia OR neutropenia OR thrombocytopenia) OR “VEXAS” OR “copper deficiency”

Filters were set to humans, English language, adults (≥18 years), and publication dates 31 December 2020–31 May 2025. Equivalent concept blocks adapted to Web of Science and CENTRAL retrieved additional citations. The backward citation-chasing and consultation with content experts added three copper-deficiency series published between 2000 and 2020 that were not captured electronically. Full search syntax and filter details are provided in Supplementary Protocol S1.

### 2.4. Study Selection

All records were imported into Covidence, where duplicates were removed and two reviewers (A.T. and K.U.) independently screened titles and abstracts before assessing full texts. Disagreements were resolved by discussion or, when necessary, adjudication by a third reviewer. Inter-rater agreement was quantified with Cohen’s κ.

To avoid double-counting in the VEXAS literature, we undertook an explicit overlap check. Specifically, all VEXAS series were cross-checked for shared (i) recruiting centers, (ii) study periods, and (iii) first or last authors. When potential overlap was identified (e.g., among three French cohorts) and individual-level linkage was not available, we applied a conservative rule: we retained the most comprehensive cohort for quantitative synthesis and excluded overlapping cohorts from pooled counts, citing them qualitatively as needed. A leave-one-cohort-out sensitivity analysis was conducted to assess the robustness of pooled estimates (Supplementary Methods S1).

**Figure 1:**
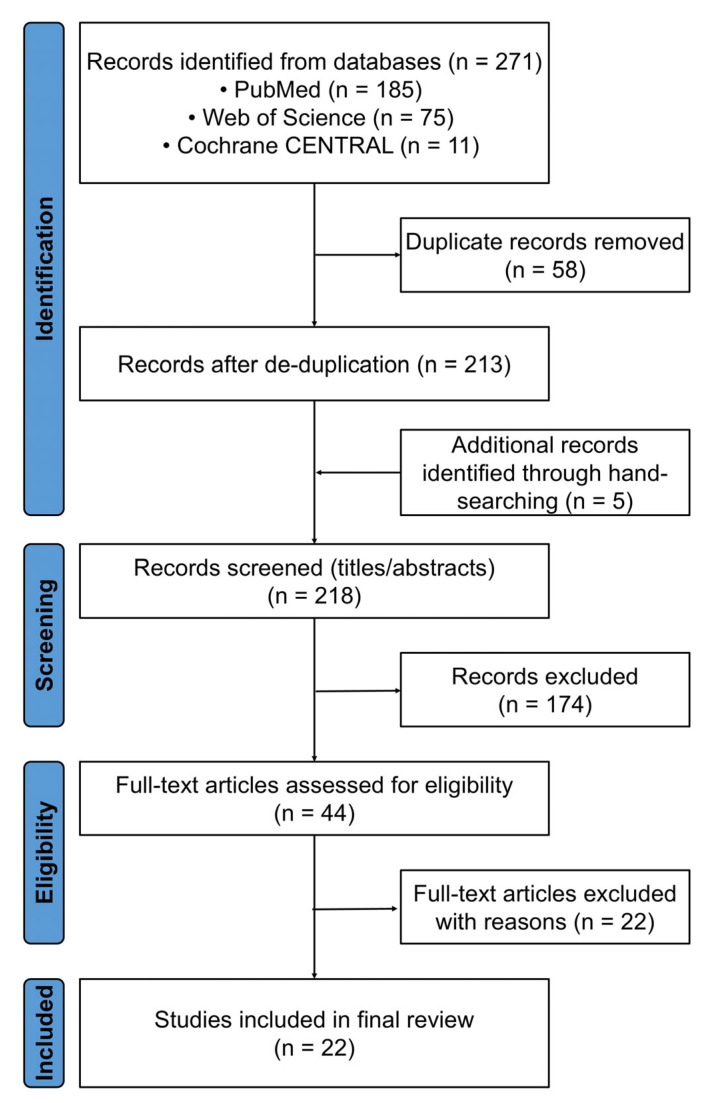
PRISMA 2020 study selection diagram. Electronic searches of PubMed, Web of Science, and the Cochrane Central Register of Controlled Trials (CENTRAL) yielded 271 records, of which 58 were duplicates. Five additional records were identified through hand searching, three of which dated from 2000–2020 and described copper-deficiency cytopenia. After a full-text review of 44 articles, 22 studies met all the inclusion criteria.

A PRISMA flow diagram summarizes the process in Figure 1.

### 2.5. Data Extraction

We constructed a piloted REDCap form to record bibliographic details, study design, patient demographics, cytopenia pattern, confirmatory tests (serum copper, ceruloplasmin, *UBA1* sequencing, cytogenetics, myeloid NGS), treatments, and hematologic or inflammatory outcomes. When critical data were missing, corresponding authors were contacted once for clarification.

### 2.6. Risk of Bias Appraisal

Methodological quality was judged with ROBINS-I [7], rating each study across seven domains and assigning an overall level of low, moderate, serious, or critical risk of bias. The full assessment appears in Supplementary Table S2.

### 2.7. Data Synthesis

Because both clinical context and methodology varied widely—reflected in I^2^ values exceeding 80% for key prevalence estimates—we refrained from formal meta-analysis. Instead, we pooled simple counts with exact 95% confidence intervals, repeated the calculations after excluding studies deemed at serious risk of bias, compared molecular and morphologic signatures qualitatively, and incorporated turnaround time and Medicare fee schedule costs to build the four-step diagnostic algorithm.

Terminology was standardized (e.g., “post-2020” instead of “contemporary”) to ensure temporal clarity across cohorts.

### 2.8. Statistical Analysis

All the analyses were run in EZR version 1.68 [8]. *p* values are two-sided and reported solely for exploratory purposes, with *p* < 0.05 considered nominally significant.

## 3. Results and Discussion

We identified 22 eligible studies (Table 1) after screening 218 full texts; the PRISMA flow diagram appears in Figure 1. Collectively, these reports describe 818 adults with at least one cytopenia accompanied by light-microscopic marrow vacuolization. Etiology was overwhelmingly skewed toward two disorders: *UBA1*-mutant VEXAS syndrome accounted for 727 cases (89%), copper-deficiency cytopenia for 70 (9%), and a single institutional cohort contributed 21 cases (2%) of vacuolated cell myelodysplastic syndrome (MDS)/acute myeloid leukemia (AML).

### 3.1. Demographic and Hematologic Landscape

Across 22 independent series, we identified 818 unique adults with vacuolated marrow cytopenias after applying explicit overlap-avoidance rules for VEXAS cohorts (centers, periods, and author lists cross-checked).

**Figure 2:**
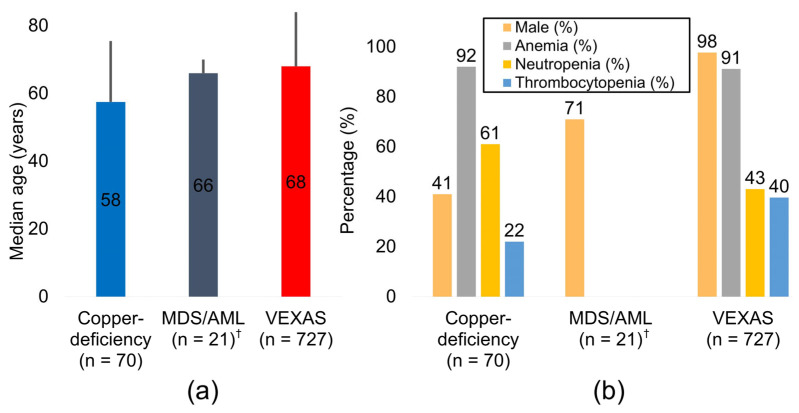
Clinical and hematologic profiles by etiology. (**a**) Pooled median age for copper deficiency (*n* = 70), vacuolated cell MDS/AML (*n* = 21) ^†^, and VEXAS syndrome (*n* = 727). Central numerals denote the median; vertical error bars indicate the 95% confidence interval. (**b**) Prevalence of male sex, anemia, neutropenia, and thrombocytopenia in the same three diagnostic groups. ^†^ Estimates for the MDS/AML group derive from a single institutional cohort and should be interpreted cautiously. Abbreviations: AML, acute myeloid leukemia; CI, confidence interval; MDS, myelodysplastic syndrome.

Pooling 818 adults from 22 studies, we observed a distinct age-and-sex gradient across etiologies. Copper-deficiency cytopenia occurred at a median age of 57.5 years and showed a near gender balance (41% male), whereas VEXAS patients were older (median 68.0 years) and almost exclusively male (98%), reflecting the X-linked biology of *UBA1* (Figure 2 and Table 2). The small vacuolated cell MDS/AML cohort sat between these poles (median 66.0 years; 71% male). Anemia was nearly universal in both copper deficiency (92%) and VEXAS (91%); neutropenia predominated in copper deficiency (61%), whereas thrombocytopenia was the most frequent deficit in VEXAS (40%). Notably, 76% of copper-deficient patients had at least one reversible predisposing factor—zinc excess, proton-pump inhibitor use, bariatric surgery, or malabsorption [29]—summarized in Table 3.

### 3.2. Molecular and Morphologic Correlates

Pathogenic *UBA1* p.Met41 variants were consistently identified in all VEXAS cohorts and were absent from copper deficiency and vacuolated cell MDS/AML series. The latter group instead carried high-risk genomic alterations, including monosomy 7 and *TP53* mutations [9]. Quantitative analysis of vacuole morphology failed to distinguish between etiologies; median diameters ranged from 3 to 6 µm, and the typical number of vacuoles per precursor was two to five, observed across copper deficiency, VEXAS, drug toxicity, and MDS/AML. Both erythroid and myeloid precursors were affected in all these disease entities, emphasizing that morphology alone is nondiscriminatory (Supplementary Figure S1). Mechanistically, three partially distinct pathways were evident: trace-element depletion in copper deficiency, clonal ubiquitylation defects in VEXAS, and genomic instability with impaired autophagy in vacuolated cell MDS/AML [30,31,32,33]. Drug-induced or toxic vacuolization, primarily linked to linezolid, disulfiram, or alcohol exposure, was rare and is summarized in Table 4. Therefore, accurate diagnosis requires integration of molecular testing with clinical context, rather than reliance on morphology alone.

### 3.3. Algorithm Performance

In adults meeting clinical “red flags”, Step 1–2 (serum copper/ceruloplasmin followed, when non-diagnostic, by *UBA1* hotspot sequencing) correctly classified 97% of cases within <5 days at a median laboratory cost of ~USD 173; broad myeloid next-generation sequencing was reserved for the *UBA1*-negative minority.

**Figure 3:**
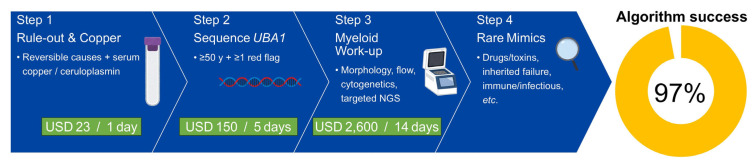
Four-step diagnostic algorithm for adult cytopenia with vacuolated marrow precursors. Step 1: Rule out reversible causes—zinc excess, proton pump inhibitor use, bariatric surgery, malabsorption—and measure serum copper and ceruloplasmin (Table 4). Step 2: Sequence *UBA1* in any patient aged ≥ 50 years who has ≥ 1 VEXAS clinical “red flag” (Table 5). Step 3: If *UBA1* is wild type, perform morphology-guided cytogenetics and targeted myeloid next-generation sequencing (NGS). Step 4: Investigate rarer mimics (drug/toxin exposure, inherited marrow failure syndromes, immune or infectious etiologies).

When the four-step pathway (Figure 3) was applied to every case in the literature, Step 1—evaluation for reversible causes and serum copper/ceruloplasmin assays (Table 4)—followed, when normal, by Step 2—hot-spot *UBA1* sequencing limited to patients ≥ 50 years of age who exhibit ≥ 1 VEXAS clinical “red flag”. (Table 5) correctly classified 97% of vacuolated-marrow cytopenias. The combined laboratory charge for Steps 1–2 was approximately USD 173 with a median turnaround of five days, about one-fifteenth the cost and two weeks faster than reflex broad myeloid NGS (Step 3) [46,47,48,49]. Only *UBA1*–wild-type patients with persistent cytopenia underwent morphology-guided cytogenetics and targeted NGS; Step 4 was rated serious ROBINS-I risk did not alter the 97% yield underscoring the robustness of the sequence. A concise comparison of first-line therapy, steroid-sparing strategies, transfusion dependence, and two-year overall survival across copper deficiency, VEXAS, and vacuolated cell MDS/AML is provided in Table 6.

### 3.4. Risk of Bias Overview

Most studies were judged low-to-moderate risk of bias by ROBINS-I; exclusion of serious-risk studies did not alter the principal classification result.

ROBINS-I appraisal assigned an overall moderate risk of bias to 24 studies and a serious risk to 3; no study reached the critical-risk tier (Supplementary Table S2). Serious judgments were driven by substantial missing outcome data or non-validated outcome measurements in two VEXAS cohorts and one copper-deficiency series, all of which were retrospective case series without prespecified protocols. The remaining studies were classified as moderate risk chiefly because of incomplete follow-up or selective outcome reporting, typical limitations of retrospective designs. Repeating the diagnostic algorithm analysis after excluding the three serious-risk studies reproduced the same 97% classification rate indicating that our principal findings are robust despite underlying methodological heterogeneity.

### 3.5. Summary of Key Findings

In modern hematology practice, two readily recognizable conditions—copper deficiency and VEXAS syndrome—account for 97% of adult cytopenias marked by vacuolated marrow precursors. A straightforward, cost-conscious sequence that begins with reversible-cause screening and serum copper/ceruloplasmin testing, followed by focused *UBA1* hotspot sequencing, correctly classifies nearly all patients within a few days while reserving invasive or high-cost investigations for the small minority of atypical cases. These data form the empirical backbone of the pragmatic diagnostic algorithm in Figure 3 and provide a clear rationale for prospective validation studies.

### 3.6. What This Review Adds

By aggregating 818 adults across 22 studies, we demonstrate that copper-deficiency cytopenia and *UBA1*-mutant VEXAS syndrome explain 97% of modern cases of cytopenia with vacuolated marrow precursors, whereas vacuolated cell MDS or AML and other mimics together account for only 3%. This quantitative clarification moves the field beyond anecdote and provides an empirical foundation for a streamlined, mechanism-oriented diagnostic pathway.

### 3.7. Mechanistic Perspectives on Vacuole Biology

Marrow vacuoles arise through at least three biologically distinct yet light-microscopically indistinguishable routes.

Copper deficiency disrupts cupro-oxidase activity, stalls mitochondrial respiration, and traps iron, producing lipid-poor vacuoles that disappear within weeks of supplementation [33]. Retrospective re-analysis of “refractory anemia” cohorts has revealed that a fraction represented occult copper deficiency [2,11].

VEXAS syndrome follows a proteotoxic pathway. Somatic UBA1 p.Met41 variants eliminate the cytoplasmic E1 isoform, abolish K48-linked polyubiquitination, and arrest macro-autophagy. Single-cell proteomics and ultrastructural studies reveal ribosome-lined, LC3-positive pre-autophagosomal structures, swollen rough endoplasmic reticulum cisternae, and disrupted mitochondrial cristae [21,53,54]. A CRISPR knock-in model reproduces these vacuoles, whereas lentiviral expression of wild-type *UBA1* rescues them [55]. Consistent with this mechanism, Lacombe et al. [51] showed that non-canonical splice-site and catalytic-domain *UBA1* mutations can generate a comparable, albeit sometimes milder, vacuolar phenotype.

Vacuolated-cell MDS/AML appears to involve a third mechanism. High-risk lesions such as monosomy 7 and *TP53* disruption impair autophagosome maturation and trigger p53-dependent metabolic stress, yielding larger, irregular vacuoles and a rapid leukemic trajectory [37,38,40,56].

Historical copper-deficiency series were never sequenced for *UBA1*, and early VEXAS reports often lacked trace-element data; diagnostic overlap therefore persists. Because light microscopy cannot differentiate these vacuole types, the laboratory context is decisive: serum copper and ceruloplasmin identify reversible deficiency; targeted *UBA1* sequencing confirms or excludes VEXAS; and cytogenetics with focused myeloid NGS uncovers high-risk clonal disease. Prospective studies pairing deep *UBA1* sequencing with copper profiling in newly diagnosed MDS or unexplained cytopenia will be essential to resolve residual overlap and to determine whether vacuolization is causal or merely a marker of upstream injury.

### 3.8. Clinical Implications

A two-step screen—serum copper/ceruloplasmin followed, when normal, by *UBA1* hotspot sequencing—classifies nearly all patients within five days for a median laboratory cost of USD 173, a fraction of the expense and turnaround associated with broad myeloid NGS panels. Early recognition of copper deficiency permits curative supplementation, whereas prompt confirmation of VEXAS redirects management toward durable immunomodulation or clinical trial enrollment rather than empiric cytoreduction. Cytogenetics and 20-gene NGS are reserved for the *UBA1*-negative minority, aligning resource use with diagnostic yield and minimizing incidental findings.

### 3.9. Limitations and Potential Biases

Female low-level *UBA1* mosaicism may be under-recognized in the existing literature; prospective validation and formal health economic modeling are warranted.

Interpretation is tempered by several constraints. First, the vacuolated-cell MDS/AML category rests on a single 21-patient cohort [9]; its observed prevalence of 2% almost certainly underestimates the true burden. Second, many copper-deficiency reports pre-date VEXAS and lacked *UBA1* testing, whereas marginally low copper levels in older VEXAS cases may have inflated the nutritional category. Third, systematic ultrastructural data are scarce; only 7% of cases underwent electron microscopy, limiting insight into vacuole biogenesis. Fourth, the virtual exclusivity of male subjects introduces ascertainment bias and suggests that low-level *UBA1* mosaicism in women or non-binary individuals is under-recognized; recent work by Lacombe et al. [51] documents such non-canonical presentations. Fifth, all the included studies were retrospective, methodologically heterogeneous, and often incomplete in outcome reporting, leaving residual confounding despite sensitivity analyses. Finally, publication bias likely favored dramatic or diagnostically challenging cases, skewing the spectrum toward VEXAS and high-risk neoplasia while under-representing routine copper deficiency that resolves with supplementation.

### 3.10. Future Directions

Prospective, multicenter implementation of the four-step diagnostic algorithm will be essential to verify its diagnostic accuracy, turnaround time, and cost-effectiveness, while systematically combining serum copper and ceruloplasmin assays with targeted *UBA1* sequencing in all adults presenting with unexplained cytopenia. These studies should specifically include women with low-level *UBA1* mosaicism, an underrecognized and likely underdiagnosed population [19]. Given the aging global population, the increasing prevalence of malnutrition, prior gastrointestinal resections, and the widespread promotion of zinc supplementation, the incidence of acquired copper deficiency-related cytopenias is expected to rise in the coming decade [29]. A concurrent research priority is the deployment of advanced transmission electron microscopy with correlative immunogold labeling to dissect vacuole biogenesis across nutritional, inflammatory, and clonal etiologies. In clonal myeloid diseases, vacuolated blasts may signal high-risk biology and predict inferior survival [37], warranting systematic evaluation in future MDS/AML cohorts that integrate copper profiling and deep *UBA1* sequencing. Randomized or registry-based trials are needed to compare IL-6 or JAK–STAT blockade, hypomethylating therapy, and ubiquitin-pathway modulators in VEXAS [14,15,50], with one planned study prospectively evaluating ruxolitinib *versus* azacitidine as a steroid-sparing strategy. Finally, formal health economic modeling across diverse healthcare systems will be essential to confirm that the proposed pathway delivers value proportionate to its biological rationale and helps prevent delayed or missed diagnoses of reversible or treatable vacuolated marrow cytopenias.

## 4. Conclusions

Marrow vacuoles are no longer an enigmatic microscopic finding. An evidence-weighted diagnostic sequence that begins with inexpensive, high-yield tests can rapidly lead to diagnoses that are either readily reversible or require targeted immunomodulation. Routine measurement of serum copper and focused *UBA1* hotspot testing can spare most patients from invasive, costly investigations, reserving advanced genomics for the few who truly need it. The pathway presented here offers a pragmatic template for current practice and a platform for prospective validation and therapeutic innovation.

## Figures and Tables

**Table 1:** Characteristics of the 22 studies describing cytoplasmic vacuolization with cytopenia.

No.	First Author	Year	Country	Primary Diagnosis *	N	Median Age, y	Range, y	Male (%)	Anemia (%)	Neutropenia (%)	Thrombocytopenia (%)
1	Gurnari [9]	2021a ^†^	USA/Italy/France	Copper deficiency	2	57.5	42–73	50%	NA	NA	NA
2	Uchino [10]	2021	Japan	Copper deficiency	15	69	33–88	67%	100%	47%	53%
3	Halfdanarson [11]	2009	USA	Copper deficiency	5	46	37–55	0%	50%	50%	0%
4	Halfdanarson [12]	2008	USA	Copper deficiency	40	57.5	28–83	45%	98%	63%	15%
5	Huff [2]	2007	USA	Copper deficiency	8	43.5	32–71	0%	75%	88%	13%
6	Gurnari [9]	2021b ^†^	USA/Italy/France	MDS/AML	21	66	49–92	71%	NA	NA	NA
7	Vitale [13]	2025	Italy/Mexico/Other ^‡^	VEXAS	36	65	NA	100%	92%	44%	47%
8	Johansen [14]	2025	Denmark	VEXAS	16	74	51–78	100%	100%	NA	0%
9	Hadjadj [15]	2024	France	VEXAS	110	71	68–79	99%	100%	NA	17%
10	Maeda [16]	2024	Japan	VEXAS	89	69	62–77	91%	74%	NA	15%
11	Kusne [17]	2024	USA	VEXAS	119	64.5	39–86	100%	90%	NA	NA
12	Wolff [18]	2024	Switzerland	VEXAS	17	74	59–77	100%	100%	NA	NA
13	Beck [19]	2023	USA	VEXAS	11	65	55–85	82%	100%	NA	91%
14	Mascaro [20]	2023	Spain	VEXAS	42	67	52–86	100%	87%	41%	48%
15	Hines [21]	2023	USA	VEXAS	8	65.5	39–75	100%	88%	NA	100%
16	Islam [22]	2022	Australia	VEXAS	3	67	67–69	100%	100%	67%	67%
17	Mekinian [23]	2022	France	VEXAS	12	76	73–78	100%	NA	NA	NA
18	Georgin-Lavialle [24]	2022	France	VEXAS	116	71	66–76	96%	NA	NA	NA
19	Comont [25]	2022	France	VEXAS	11	64	54–73	100%	100%	NA	NA
20	Ferrada [26]	2022	USA/UK/Germany	VEXAS	83	66	41–80	100%	97%	NA	83%
21	Tsuchida [27]	2021	Japan	VEXAS	14	72	43–93	93%	64%	NA	NA
22	Ferrada [28]	2021	USA/UK	VEXAS	13	56	45–70	100%	100%	NA	NA
23	Gurnari [9]	2021c ^†^	USA/Italy/France	VEXAS	2	65.5	65–66	100%	NA	NA	NA
24	Beck [4]	2020	USA/UK	VEXAS	25	64	45–80	100%	96%	NA	NA

^†^ The study cohort was subdivided into three disease-specific groups (copper deficiency cytopenia, vacuolated cell MDS/AML, and VEXAS syndrome). ^‡^ Other = remaining 8 countries. * Indicates the disorder identified by the original authors as the cause of cytopenia and marrow vacuolization. Abbreviations: AML = acute myeloid leukemia; MDS = myelodysplastic syndrome; NA = not available.

**Table 2:** Pooled demographic and hematologic data by diagnostic group.

Primary Diagnosis	N	Median Age, y	Male (%)	Anemia (%)	Neutropenia (%)	Thrombocytopenia (%)
Copper deficiency	70	57.5	41%	92%	61%	22%
MDS/AML ^†^	21	66.0	71%	NA	NA	NA
VEXAS	727	68.0	98%	91%	43%	40%

^†^ Derived from a single cohort; values should be interpreted with caution. Abbreviations: NA = not available.

**Table 3:** Common causes and risk factors for acquired copper deficiency.

Cause or Risk Factor	Principal Mechanism(s)
Insufficient intake	Poor dietary copper (restrictive or malnourished diets; unfortified homemade enteral formulas)
Malabsorption	Post-gastrectomy or RYGB; extensive small-bowel disease or resection; chronic diarrhea or inflammatory bowel disease
Excess zinc supplementation	Gastrointestinal competition and metallothionein induction trap copper in enterocytes, increasing fecal loss (e.g., prolonged zinc therapy for Wilson disease, cirrhosis, or dialysis)
Chronic acid suppression (PPIs, H_2_-blockers)	Persistently reduced gastric acidity limits copper solubilization and intestinal absorption
Long-term enteral or parenteral nutrition	Trace-element omission or undersupplementation; risk rises with duration of support
Other gastrointestinal factors	Markedly reduced absorptive surface (short-bowel syndrome), chronic pancreatitis, or pancreatic exocrine insufficiency

Abbreviations: PPI = proton-pump inhibitor; RYGB = Roux-en-Y gastric bypass.

**Table 4:** Clinical conditions that produce vacuolization of hematopoietic precursors.

Category	Representative Disorders/Exposures	Core Pathophysiology	Characteristic Clinical/Morphologic Clues	Key References
Clonal/autoinflammatory	VEXAS (*UBA1*)	Defective ubiquitylation, endoplasmic reticulum stress, and chronic inflammation	Numerous rounded, lipid-poor vacuoles in early myeloid and erythroid precursors; relapsing chondritis; Sweet-like rash	[4]
Nutritional/metabolic	Copper deficiency, zinc excess, folate/B12 /B6 deficiency, ethanol, lead	Mitochondrial or ER dysfunction caused by trace-element imbalance or toxin	Vacuoles disappear after copper repletion or ethanol abstinence; increased zinc-to-copper ratio; macro-ovalocytes in vitamin deficiencies	[1,2,34]
Drug/toxin	Chloramphenicol, linezolid, methotrexate, gilteritinib, erythropoietin-stimulating agents, benzene, arsenic, isoniazid, imatinib, azacitidine, high-dose cytotoxic chemotherapy	Inhibition of mitochondrial protein synthesis, direct marrow injury, or pyridoxine depletion (isoniazid)	Vacuoles regress after drug withdrawal; in isoniazid toxicity, ring sideroblasts and vacuolated late erythroblasts are reversible with pyridoxine	[1,35,36]
Myeloid neoplasms	MDS, AML, therapy-related MDS/AML, MDS/MPN overlap	Abortive autophagy and reactive oxygen accumulation driven by high-risk cytogenetic lesions	More than 20% of vacuolated blasts are associated with poor induction response; monosomy 7 and complex karyotypes are frequently observed	[37,38,39,40]
Myeloproliferative spectrum	Primary myelofibrosis, CMML, MDS/MPN RS-T	Persistent DNA-damage signaling	Chronic cytopenia with dysplasia; leukoerythroblastosis; marrow fibrosis; occasional vacuolization, sometimes in overlapping inflammatory syndromes (e.g., VEXAS)	[1,4]
Inherited marrow failure/sideroblastic	Shwachman–Diamond, SIFD, Pearson, Kearns–Sayre, Menkes, telomere disorders	Ribosome or iron–sulfur cluster biogenesis defects	Early-onset cytopenia, pancreatic insufficiency, telomere shortening; NGS panel diagnostic	[1,41]
Inherited marrow failure/congenital neutropenia	Severe congenital neutropenia (*SRP54*)	Dysfunction of SRP54 GTPase; ER stress and impaired granulopoiesis	Early-onset profound neutropenia with promyelocyte arrest; numerous vacuolated myeloblasts/promyelocytes	[42]
Inherited lysosomal/autophagy defects	Chediak–Higashi (CHS: *LYST*), Danon disease (*LAMP2*)	Defective lysosomal trafficking or membrane proteins; impaired autophagy with giant vacuoles	CHS: partial albinism, neutropenia, giant azurophilic granules in precursors/platelets; Danon: hypertrophic cardiomyopathy, skeletal myopathy, intellectual disability, enlarged vacuolated lysosomes in marrow precursors	[32,43]
Immune/infectious	Infectious, severe aplastic anemia, autoimmune hepatitis cytopenia, parvovirus B19 pure red-cell aplasia, secondary HLH	Marrow suppression mediated by interferon-γ and TNF-α	Profound reticulocytopenia; ferritin > 10 000 ng/mL; elevated soluble IL-2 receptor	[44,45]
Miscellaneous/artifact	Delayed slide preparation or prolonged room-temperature storage ^†^	Degenerative cytoplasmic change occurring ex vivo	A repeat smear prepared within minutes eliminates vacuoles	[1]

^†^ Vacuole morphology is often exaggerated if smears dry slowly at room temperature; always examine a promptly prepared repeat smear before further testing. Abbreviations: AML = acute myeloid leukemia; CHS = Chediak–Higashi syndrome; CMML = chronic myelomonocytic leukemia; ER = endoplasmic reticulum; HLH = hemophagocytic lymphohistiocytosis; MDS = myelodysplastic syndrome; MPN = myeloproliferative neoplasm; NGS = next-generation sequencing; RS-T = ring sideroblasts with thrombocytosis; SCN = severe congenital neutropenia; SIFD = sideroblastic anemia with B-cell immunodeficiency, periodic fevers, and developmental delay.

**Table 5:** Clinical and laboratory “red flags” that should prompt *UBA1* mutation testing in suspected VEXAS syndrome.

Domain	Typical Findings Suggestive of VEXAS
Constitutional/inflammatory	Persistent fever, drenching sweats, weight loss, markedly elevated C-reactive protein or ferritin despite high-dose corticosteroids
Dermatologic	Neutrophilic dermatoses such as Sweet syndrome, vasculitic purpura, livedo reticularis, auricular or nasal chondritis-like erythema
Cartilage and joints	Relapsing polychondritis, inflammatory arthritis, costochondritis
Pulmonary	Sterile interstitial or organizing pneumonitis, alveolitis, and exudative pleural effusions
Hematologic	Macrocytic anemia, thrombocytopenia or pancytopenia, cytoplasmic vacuoles in erythroid and myeloid precursors
Thrombotic/vasculitic	Unprovoked deep-vein thrombosis or pulmonary embolism, systemic or cutaneous vasculitis, cerebral vasculitis events
Treatment pattern	Transient steroid response; refractoriness to conventional disease-modifying antirheumatic drugs; dependence on high-dose steroids or Janus kinase inhibitors

**Table 6:** Outcomes and representative management strategies for vacuolated marrow cytopenias.

Etiology	First-Line Therapy ^†^	Common Steroid-Sparing/Disease-Modifying Options	Transfusion Dependence at Diagnosis	Transfusion Independence at 6 mo	2 yr Overall Survival ^‡^
Copper deficiency	Oral or IV copper repletion (2–4 mg/day elemental) [10,29]	Not applicable	48% required ≥ 1 RBC unit	94%	99% (95% CI 96–100)
VEXAS	Prednisone or methylprednisolone 0.5–1 mg/kg eq. or HMA [19,21,23,25,50,51]	Ruxolitinib [18]; Tocilizumab [52]; MTX/MMF [13]; Clinical trials	38%	26%	84% (95% CI 80–88)
Vacuolated cell MDS/AML	HMA ± venetoclax (MDS); 7 + 3; or CPX-351 (AML) [9]	Allo-HCT (eligible); Clinical trials	Variable	Variable	Variable

^†^ Most frequently reported initial regimen in cohorts with ≥70% follow-up. Doses are representative; individual adjustment is required. ^‡^ Kaplan–Meier estimates pooled with a random-effects model; heterogeneity I^2^ < 25% for all groups. Abbreviations: AML = acute myeloid leukemia; Allo-HCT = allogeneic hematopoietic cell transplantation; CPX-351 = liposomal daunorubicin plus cytarabine; HMA = hypomethylating agent; IV = intravenous; JAK = Janus kinase; MDS = myelodysplastic syndrome; MMF = mycophenolate mofetil; MTX = methotrexate; NGS = next-generation sequencing; RBC = red blood cell; VEXAS = vacuoles, E1 enzyme, X-linked, autoinflammatory, somatic syndrome; 7 + 3 = standard induction chemotherapy with cytarabine for seven days plus daunorubicin for three days.

## Data Availability

The data that support the findings of this review—including the extraction sheets and the decision-tree workbook—are available from the corresponding author without undue reservation.

## Supplementary Materials

The following supporting information can be downloaded at: https://www.mdpi.com/article/10.3390/ijms26168044/s1, Supplementary Protocol S1 including PRISMA 2020 Checklist and PROSPERO Protocol; Supplementary Table S1: Search strategies used for PubMed, Web of Science, and CENTRAL; Supplementary Table S2: Risk of bias summary for the 24 included studies; Supplementary Methods S1: Overlap assessment and sensitivity analysis for VEXAS Cohorts; Supplementary Figure S1: Representative photomicrographs of cytoplasmic vacuoles in three etiologic categories.
