# Supplementary material for: Vacuolated Marrow Cytopenias from Copper Deficiency to UBA1-Mutant VEXAS: Molecular Landscape, Systematic Review, and Cost-Efficient Diagnostic Algorithm
Source: Int J Mol Sci. 2025 Aug 20;26(16):8044. doi: 10.3390/ijms26168044 (PMC12386339; doi:10.3390/ijms26168044)
Supplement: Supplementary file 1 [file ijms-26-08044-s001.zip › Supplementary Figure S1.pdf]

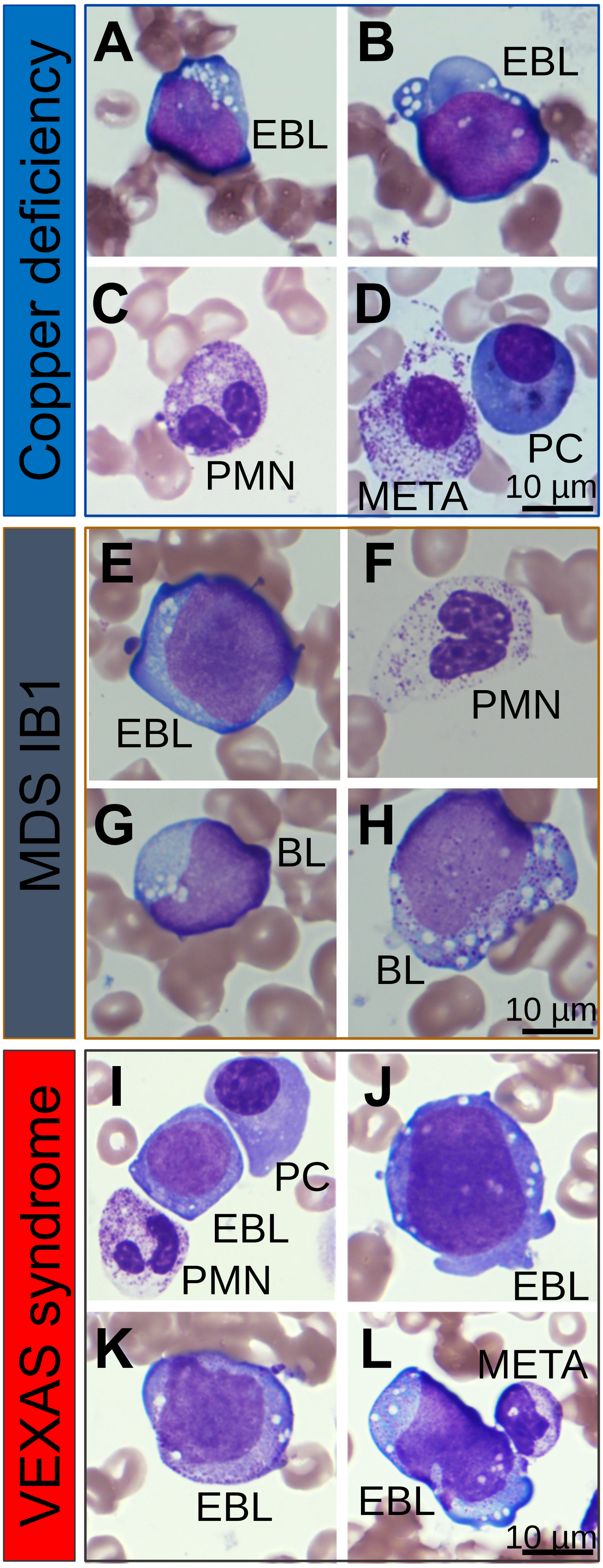

Supplementary Figure S1. Representative photomicrographs of cytoplasmic vacuoles in three etiologic categories. May–Giemsa stain; bright field, oil immersion × 1,000; scale bar = 10 µm. Copper deficiency cytopenia (A–D): (A, B) vacuolated erythroblasts (EBL); (C) vacuolated neutrophil (PMN); (D) vacuolated metamyelocyte (META) adjacent to a hemosiderin laden plasma cell (PC). MDS IB1 (E–H): (E) vacuolated EBL; (F) vacuolated PMN; (G, H) vacuolated blasts (BL) amid dysplasia. VEXAS syndrome (I–L): (I) vacuolated EBL with accompanying PMN and PC; (J-L) additional vacuolated EBL; (L) vacuolated META. All smears were collected under an institutional review board protocol (Aichi Medical University #2021-022) and are fully deidentified. Abbreviations: BL, blast; EBL, erythroblast; META, metamyelocyte; PC, plasma cell; PMN, polymorphonuclear neutrophil.
