# Supplementary material for: Vacuolated Marrow Cytopenias from Copper Deficiency to UBA1-Mutant VEXAS: Molecular Landscape, Systematic Review, and Cost-Efficient Diagnostic Algorithm
Source: Int J Mol Sci. 2025 Aug 20;26(16):8044. doi: 10.3390/ijms26168044 (PMC12386339; doi:10.3390/ijms26168044)
Supplement: Supplementary file 1 [file ijms-26-08044-s001.zip › Supplementary Methods_S1.pdf]

Supplementary Methods S1. Overlap Assessment and Sensitivity Analysis for VEXAS Cohorts

S1.1 Identification of Potentially Overlapping Cohorts

To prevent double counting of individual patients across VEXAS series, we evaluated every included study for three overlap indicators:

1. Recruiting center(s) – identical hospital or national registry.
2. Study period – overlapping calendar years of patient enrollment.
3. Authorship – identical first or last author, or shared senior collaborators.

A cohort was flagged as "potentially overlapping" if it matched another study on at least two of these three criteria. Using this rule, three French VEXAS cohorts (designated A, B, and C) were flagged.

S1.2 Conservative Inclusion Rule

For each flagged group, we retained the most comprehensive dataset—defined hierarchically as the cohort with (i) the longest enrollment window, then (ii) the largest sample size, then (iii) the greatest breadth of clinical variables. Redundant cohorts were excluded from quantitative synthesis but cited qualitatively where unique phenotypic details were reported.

S1.3 Leave-One-Cohort-Out Sensitivity Analysis

To assess the robustness of pooled proportions and algorithm performance, we performed a leave-one-cohort-out (LOCO) analysis that iteratively removed each VEXAS cohort (including the retained French cohort A) and recalculated:

- The pooled frequency of VEXAS among all vacuolated cytopenias (DerSimonian–Laird random-effects model).
- The step-specific sensitivity and specificity of the four-step diagnostic algorithm.

Calculations were executed by EZR version 1.68. Heterogeneity was expressed as I^2.

S1.4 Results of the Sensitivity Analysis

• Pooled VEXAS frequency: Base-case estimate = 88.9% (95% CI 85.6–92.2%). Across LOCO iterations the estimate ranged from 87.8% to 90.1%; I^2 changed by < 3%.

• Algorithm accuracy: Base-case overall accuracy = 97.1%. LOCO values ranged from 96.8% to 97.4%. No single cohort shifted sensitivity or specificity by more than 0.6 percentage points.

S1.5 Interpretation

The minimal variation observed in LOCO analyses indicates that possible patient overlap does not materially influence the pooled prevalence of VEXAS or the performance metrics of

the diagnostic algorithm. Therefore, our conservative inclusion strategy adequately mitigates the risk of double counting while preserving statistical robustness.
