# Supplementary material for: Vacuolated Marrow Cytopenias from Copper Deficiency to UBA1-Mutant VEXAS: Molecular Landscape, Systematic Review, and Cost-Efficient Diagnostic Algorithm
Source: Int J Mol Sci. 2025 Aug 20;26(16):8044. doi: 10.3390/ijms26168044 (PMC12386339; doi:10.3390/ijms26168044)
Supplement: Supplementary file 1 [file ijms-26-08044-s001.zip › Supplementary Protocol S1.pdf]

Supplementary Protocol S1

Prespecified Protocol for the Systematic Review "Vacuolated-Marrow Cytopenias in Adults: A Systematic Review, Institutional Case Series, and Pragmatic Diagnostic Algorithm."

1 Background and Rationale

Cytoplasmic vacuolization of hematopoietic precursors (HPs) has long been viewed as a nonspecific dysplastic change. Recent discoveries—especially copper-deficiency–related cytopenia and UBA1-mutated VEXAS syndrome—suggest that the finding is far more specific and clinically actionable than previously believed. A rigorous synthesis of the contemporary evidence is therefore warranted to guide diagnostic sequencing and therapeutic decision-making.

2 Objectives

1. Estimate the relative frequencies of copper deficiency, VEXAS syndrome, and clonal myeloid neoplasms among adults presenting with vacuolated-marrow cytopenia.

2. Summarize clinical, hematologic, molecular, and treatment-response profiles for each aetiology.

3. Develop an evidence-anchored, workflow-compatible diagnostic algorithm that prioritizes reversible and highly treatable causes.

4. Identify knowledge gaps—including a residual category of unexplained cases—that merit future genomic interrogation.

3 Registration

The review is registered in PROSPERO (CRD420251082738). This registration was updated on 3 July 2025 to reflect refinements in search strategy and eligibility criteria. The full protocol remains available as Supplementary Protocol S1.

4 Eligibility Criteria (PICO)

Domain	Inclusion	Exclusion

**Population**	Adults ≥18 y with ≥1 cytopenia (Hb <12 g/dL, ANC <1.5×10^9/L, or platelets <150×10^9/L) and light-microscopic evidence of cytoplasmic vacuoles in marrow-precursors.	Pediatric cohorts; animal or in-vitro studies.
**Intervention / Exposure**	Not applicable (observational synthesis).	
**Comparators**	None required; descriptive across diagnostic groups.	
**Outcomes**	(i) Prevalence of each diagnostic category; (ii) age, sex, and cytopenia patterns; (iii) molecular/cytogenetic findings; (iv) hematologic and inflammatory responses to therapy.	
**Study Designs**	Cohort, cross-sectional, or case-series reports that include ≥2 eligible adults.	Single-case reports, conference abstracts without full data, narrative reviews, editorials.
**Language**	English.	
**Time Frame**	Primary search: 31 Dec 2020 – 3 Jul 2025. Historical copper-deficiency cohorts (2000 – 2020) manually added.	

5 Information Sources

- Electronic databases: PubMed/MEDLINE, EMBASE (Elsevier), Web of Science (Clarivate), and Cochrane CENTRAL.

- **Hand-searching**: reference lists of included studies; expert recommendations.

- **Institutional registry**: Aichi Medical University marrow-failure registry queried for 2023 – 2025.

6 Search Strategy

Full, line-by-line PubMed query:

("VEXAS"[All Fields] OR "copper deficiency"[Title/Abstract] OR

(("pancytopenia"[Title/Abstract] OR "cytopenia"[Title/Abstract]) AND

("bone marrow"[Title/Abstract] AND

("vacuole"[Title/Abstract] OR "vacuoles"[Title/Abstract] OR "vacuolization"[Title/Abstract]))))

AND ("adult"[MeSH Terms] OR "adult"[Title/Abstract])

AND ("2020/12/31"[PDAT] : "2025/05/31"[PDAT])

AND (english[Language])

Equivalent strategies for EMBASE and Web of Science are detailed in Supplementary Table S1.

7 Study Selection

Records will be imported into EndNote X9 and de-duplicated. Two reviewers (A.T., K.U.) will independently screen titles and abstracts. Full texts will be obtained for any record that is (i) explicitly relevant or (ii) ambiguous. Disagreements will be resolved by consensus; a third reviewer will adjudicate if needed. A PRISMA 2020 flow diagram will document the process.

8 Data Extraction

A pilot-tested spreadsheet (Excel 365) will capture:

- Bibliographic details, country, and study design.

- Cohort size; median and range of age.

- Sex distribution.

- Prevalence of anemia, neutropenia/leukopenia, thrombocytopenia.

- Diagnostic confirmation (serum copper, ceruloplasmin, UBA1 sequencing, cytogenetics, NGS panel).

- Therapeutic interventions and hematologic/inflammatory outcomes when reported.

Two reviewers will extract in duplicate; discrepancies will be reconciled through discussion.

9 Risk-of-Bias Assessment

Because all eligible studies are observational, the ROBINS-I tool will be applied, focusing on three prespecified domains most relevant to descriptive frequency

estimates—selection, misclassification, and attrition. Each domain will be graded as low, moderate, or high risk of bias; results will be visualized in Supplementary Table S2.

10 Data Synthesis

Given anticipated clinical and methodological heterogeneity, a formal meta-analysis is not planned. Instead, pooled medians (age) and pooled proportions (cytopenia patterns) will be calculated where ≥3 studies contribute data; no statistical weighting will be applied. Findings will be narratively compared across diagnostic categories.

10.1 Algorithm Construction

The stepwise diagnostic algorithm will be derived by mapping (i) prevalence of each cause, (ii) median turnaround and cost of diagnostic tests, and (iii) treatability/reversibility, prioritizing tests that maximize early, actionable diagnoses.

11 Sensitivity and Subgroup Analyses

If ≥2 large (n≥50) VEXAS cohorts are available, we will compare outcomes in patients who did versus did not receive disease-modifying agents. If insufficient data, analysis will remain descriptive.

12 Confidence in Cumulative Evidence

We will apply a simplified GRADE approach assessing risk of bias, inconsistency, indirectness, imprecision, and publication bias.

13 Protocol Amendments

Any deviations from this protocol will be transparently reported in the final manuscript and in **Supplementary Table S2**.

14 Ethics and Dissemination

No primary patient data are collected; ethical approval is unnecessary. Results will be submitted to a peer-reviewed hematology journal and presented at the American Society of Hematology annual meeting.

Protocol finalized: 30 May 2025.

Corresponding author: Akiyoshi Takami, MD (takami-knz@umin.ac.jp)

NIHR | National Institute for Health and Care Research

PROSPERO
International prospective register of systematic reviews

Vacuolated-Marrow Cytopenias in Adults: A Systematic Review, Institutional Case Series, and Pragmatic Diagnostic Algorithm

Kaori Uchino, Megumi Enomoto

Citation
Kaori Uchino, Megumi Enomoto. Vacuolated-Marrow Cytopenias in Adults: A Systematic Review, Institutional Case Series, and Pragmatic Diagnostic Algorithm. PROSPERO 2025 CRD420251082738. Available from https://www.crd.york.ac.uk/PROSPERO/view/CRD420251082738.

REVIEW TITLE AND BASIC DETAILS

Review title
Vacuolated-Marrow Cytopenias in Adults: A Systematic Review, Institutional Case Series, and Pragmatic Diagnostic Algorithm

Condition or domain being studied
Autoinflammatory disease; Myelodysplastic Syndrome; Copper deficiency; Bone Marrow Finding; Cytopenia; Acute Myelocytic Leukemia

Rationale for the review
Cytoplasmic vacuolization of hematopoietic precursors (HPs) has long been viewed as a nonspecific dysplastic change. Recent discoveries—especially copper-deficiency–related cytopenia and UBA1-mutated VEXAS syndrome—suggest that the finding is far more specific and clinically actionable than previously believed. A rigorous synthesis of the contemporary evidence is therefore warranted to guide diagnostic sequencing and therapeutic decision-making.

Review objectives
1. Estimate the relative frequencies of copper deficiency, VEXAS syndrome, and clonal myeloid neoplasms among adults presenting with vacuolated-marrow cytopenia.

2. Summarize clinical, hematologic, molecular, and treatment-response profiles for each aetiology.

3. Develop an evidence-anchored, workflow-compatible diagnostic algorithm that prioritizes reversible and highly treatable causes.

4. Identify knowledge gaps—including a residual category of unexplained cases—that merit future genomic interrogation.

Keywords

Bone Marrow Aspirate; VEXAS syndrome; MDS; AML; UBA1 mutation; Cytopenia

Country

Japan

ELIGIBILITY CRITERIA

Population

Included
Studies were eligible if they:

enrolled adults (≥ 18 y) with one or more cytopenias;

documented cytoplasmic vacuoles in hematopoietic precursors on bone-marrow aspirate or biopsy; and

reported at least hemoglobin, neutrophil (or leukocyte), and platelet counts.

Excluded
We excluded single-case reports, pediatric series, narrative reviews without primary data, and studies describing purely transient or drug-induced vacuolization.

Intervention(s) or exposure(s)

Included
Observational Assessment

Comparator(s) or control(s)

This review does not have any comparators

Study design

Only nonrandomized study types will be included.

Context

Eligible studies must involve adult patients (≥ 18 years) evaluated for one or more cytopenias in secondary- or tertiary-care medical settings (e.g., university hospitals, specialised haematology/oncology centres, or referral pathology laboratories).

• The index finding—cytoplasmic vacuoles in bone-marrow precursors—must be documented on aspirate smears or trephine biopsies reviewed by qualified haematopathologists.

• Studies from any geographic region or income level are eligible, provided they are published in English-language, peer-reviewed journals between 2000 and 2025.

• We will exclude reports limited to paediatric patients, veterinary or animal models, purely in-vitro investigations, conference abstracts without full data, and single-patient case reports.

TIMELINE OF THE REVIEW

Date of first submission to PROSPERO
29 June 2025

Review timeline
Start date: 1 June 2025. End date: 30 September 2025.

Date of registration in PROSPERO
30 June 2025

AVAILABILITY OF FULL PROTOCOL

Availability of full protocol
A full protocol has been written and uploaded to PROSPERO. The protocol will be made available after the review is completed.

SEARCHING AND SCREENING

Search for unpublished studies
Only published studies will be sought.

Main bibliographic databases that will be searched
The main databases to be searched are *Embase.com*, *PubMed* and *SCI - Science Citation Index*.

Search language restrictions
The review will only include studies published in English.

Search date restrictions
Databases will be searched for articles published from 31 December 2020 and before by 31 May 2025.

Other methods of identifying studies
Other studies will be identified by: *contacting authors or experts* and *reference list checking*.

Additional information about identifying studies
Limiting database searches to 2020 onwards may overlook earlier vacuolated-marrow cytopenia reports not labeled as copper deficiency or VEXAS. However, key pre-2020 copper-deficiency cohorts were hand-searched, and our tiered strategy preserves specificity while reducing noise.

Link to search strategy
A full search strategy has been uploaded to PROSPERO. The PDF may be accessed through this link
https://www.crd.york.ac.uk/PROSPEROFILES/cb802a15a3f34dacca2b3dc2caa93eca.pdf.

Selection process

Studies will be screened independently by at least two people (or person/machine combination) with a process to resolve differences.

Other relevant information about searching and screening

Records are imported into EndNote 20 and de-duplicated. Two reviewers (Akiyoshi Takami, MD, PhD and Kaori Uchino, MD, PhD) independently screen titles/abstracts and then full texts against predefined criteria; disagreements are resolved by consensus or third-party adjudication. Reasons for exclusion at the full-text stage will be recorded, and the process will be summarised in a PRISMA 2020 flow diagram.

DATA COLLECTION PROCESS

Data extraction from published articles and reports

Data will be extracted independently by at least two people (or person/machine combination) with a process to resolve differences.

Authors will be asked to provide any required data not available in published reports.

Study risk of bias or quality assessment

Risk of bias will be assessed using: *ROBINS-I*

Data will be assessed independently by at least two people (or person/machine combination) with a process to resolve differences.

Additional information will be sought from study investigators if required information is unclear or unavailable in the study publications/reports.

Reporting bias assessment

If $\geq$ 10 studies contribute to a comparable outcome, we will inspect funnel plots and apply Egger's regression test (p < 0.10 suggests small-study effects). For outcomes with < 10 studies or where meta-analysis is not feasible, we will qualitatively assess reporting bias by comparing published reports with trial registries, conference abstracts, and protocols (when available) and by contacting corresponding authors for missing outcome data. Any concerns will be fed into the GRADE "publication bias" domain.

Certainty assessment

We will apply the GRADE approach to rate the certainty (quality) of evidence for each prespecified outcome.

Two reviewers will independently grade the body of evidence across the five standard GRADE domains— risk of bias (ROBINS-I results), inconsistency (I^2/overlap of CIs), indirectness (population, intervention, comparator, outcome), imprecision (95 % CI width and optimal information size), and publication bias (visual inspection of funnel plots or qualitative signals).

Starting at "high" certainty for observational evidence upgraded by large effects is not appropriate; therefore all bodies of evidence will begin at "low" and may be downgraded (-1 or -2) for serious or very serious concerns in any domain, or upgraded if a large, consistent effect with dose–response is observed.

Disagreements will be resolved by discussion or third-party adjudication. Final ratings—high, moderate, low, or very low—will be summarised in a GRADE evidence profile

(Supplementary Table S3) and used to contextualise the strength of any clinical recommendations.

OUTCOMES TO BE ANALYSED

Main outcomes

Proportion of cases attributable to (i) copper-deficiency cytopenia, (ii) UBA1-mutant VEXAS syndrome, (iii) vacuolated-marrow MDS/AML, or (iv) other causes.

Additional outcomes

Demographics (age, sex) by aetiology

Prevalence of each cytopenia (Hb < 12 g/dL, ANC < 1.5 × 10^9/L, Plt < 150 × 10^9/L)

Molecular or cytogenetic abnormalities (e.g., TP53, complex karyotype)

Hematologic or inflammatory response to therapy, where available

Overall or event-free survival when reported.

PLANNED DATA SYNTHESIS

Strategy for data synthesis

Descriptive statistics (counts, percentages, medians, ranges) will be generated in EZR v1.68. Between-study heterogeneity will be assessed with I^2; if I^2 < 60 % and ≥ 3 homogeneous cohorts report the same outcome, a random-effects meta-analysis will be considered. Otherwise, results will be narratively synthesised and presented in tabular and graphical form (stacked bar charts).

Risk of bias will be assessed with an adapted ROBINS-I (selection, misclassification, attrition), and certainty of evidence summarised using GRADE. Findings will inform a stepwise diagnostic algorithm.

CURRENT REVIEW STAGE

Stage of the review at this submission 1 change

Review stage	Started	Completed
Pilot work	✓	✓
Formal searching/study identification	✓	✓
Screening search results against inclusion criteria	✓	✓
Data extraction or receipt of IPD	✓	✓
Risk of bias/quality assessment	✓	✓
Data synthesis	✓	✓

Review status

The review is completed.

Publication of review results

Results of the review will be published in English.

REVIEW AFFILIATION, FUNDING AND PEER REVIEW

Review team members

Associate Professor Kaori Uchino (review guarantor and contact) ORCID: 0000-0003-2983-2129. Aichi Medical University. Japan.

No conflict of interest declared.

Ms Megumi Enomoto. Aichi Medical University. Japan.

No conflict of interest declared.

Named contact

Associate Professor Kaori Uchino (ksakai@aichi-med-u.ac.jp). ORCID: 0000-0003-2983-2129. Aichi Medical University. Japan.

Review affiliation

Division of Hematology, Aichi Medical University, Nagakute, Japan.

Funding source

Review has no specific/external funding but is supported by guarantor/review team (non-commercial) institutions.

Additional information about funding

No external funding; review conducted with departmental resources

Peer review

Internal review by the Aichi Medical University Hematology faculty; no external peer-review to date.

ADDITIONAL INFORMATION

Additional information

This review forms part of an integrated research programme to improve the diagnostic work-up of cytopenic adults with bone-marrow vacuolisation. Alongside the systematic review, we continue to enrol a prospective, single-centre case series; these institutional data will be reported descriptively only and will not be pooled with published studies.

The protocol was conceived in February 2025. Initial database searches were completed on 1 June 2025, but pilot screening revealed that several eligible studies were missed. Consequently, on 3 July 2025 we refined the Boolean operators, added CENTRAL, and extended the date range to ensure comprehensive capture; no study-level data extraction or ROBINS-I appraisal had been performed before this amendment, so the timing requirements remain satisfied.

Any further protocol changes (e.g., subgroup analyses) will be logged in the "History" tab. The review is unfunded, the authors declare no conflicts of interest, and results will first be

submitted to a peer-reviewed, high-impact haematology journal; subsequent submissions will be listed in this PROSPERO record as needed.

Review conflict of interest

Declared individual interests are recorded under team member details.. No additional interests are recorded for this review.

Medical Subject Headings

Adult; Algorithms; Bone Marrow; Copper; Cytopenia; Genomics; Humans; VEXAS syndrome; Workflow; Neoplasms; Myelodysplastic Syndromes; Vacuoles; Hematopoietic Stem Cells; Ubiquitin-Activating Enzymes; Anemia, Macrocytic; Diagnosis, Differential; Leukemia, Myeloid, Acute

Revision note 1 change

All review steps including data extraction, risk of bias assessment, and data synthesis were completed as planned. No significant protocol deviations occurred. We will update the record when it is published.

SIMILAR REVIEWS

Check for similar records already in PROSPERO

PROSPERO identified a number of existing PROSPERO records that were similar to this one (last check made on 28 June 2025). These are shown below along with the reasons given by that the review team for the reviews being different and/or proceeding.

- Involvement of myeloid-derived suppressor cells in bone marrow failure syndromes and other cytopenias [published 25 July 2024] [CRD42024565547]. The review was judged **not to be similar**
- Diagnostic performance and clinical and social utility of genomic autopsy following unexplained stillbirth. [published 4 May 2022] [CRD42022318765]. The review was judged **not to be similar**
- Kindler syndrome : A systemic review of published case reports and case series [published 25 June 2025] [CRD420251076633]. The review was judged **not to be similar**

PROSPERO version history 1 change

- Version 1.5, published 03 Jul 2025
- Version 1.4, published 03 Jul 2025
- Version 1.3, published 03 Jul 2025
- Version 1.2, published 30 Jun 2025
- Version 1.1, published 30 Jun 2025
- Version 1.0, published 30 Jun 2025

Disclaimer

The content of this record displays the information provided by the review team. PROSPERO does not peer review registration records or endorse their content.

PROSPERO accepts and posts the information provided in good faith; responsibility for record content rests with the review team. The guarantor for this record has affirmed that the information provided is truthful and that they understand that deliberate provision of inaccurate information may be construed as scientific misconduct.

PROSPERO does not accept any liability for the content provided in this record or for its use. Readers use the information provided in this record at their own risk.

Any enquiries about the record should be referred to the named review contact

PRISMA 2020 Checklist

Section and Topic	Item #	Checklist item	Location where item is reported
**TITLE**			
Title	1	Identify the report as a systematic review.	Title page, line 1 — “Vacuolated Marrow … Systematic Review …”
**ABSTRACT**			
Abstract	2	See the PRISMA 2020 for Abstracts checklist.	Structured Abstract (p. 1)
**INTRODUCTION**			
Rationale	3	Describe the rationale for the review in the context of existing knowledge.	1. Introduction, para 1–2
Objectives	4	Provide an explicit statement of the objective(s) or question(s) the review addresses.	1. Introduction, final paragraph
**METHODS**			
Eligibility criteria	5	Specify the inclusion and exclusion criteria for the review and how studies were grouped for the syntheses.	4.2 Eligibility criteria
Information sources	6	Specify all databases, registers, websites, organisations, reference lists and other sources searched or consulted to identify studies. Specify the date when each source was last searched or consulted.	4.3 Information sources & search strategy
Search strategy	7	Present the full search strategies for all databases, registers and websites, including any filters and limits used.	Supplementary Table S1 + 4.3
Selection process	8	Specify the methods used to decide whether a study met the inclusion criteria of the review, including how many reviewers screened each record and each report retrieved, whether they worked independently, and if applicable, details of automation tools used in the process.	4.4 Study selection
Data collection process	9	Specify the methods used to collect data from reports, including how many reviewers collected data from each report, whether they worked independently, any processes for obtaining or confirming data from study investigators, and if applicable, details of automation tools used in the process.	4.5 Data extraction
Data items	10a	List and define all outcomes for which data were sought. Specify whether all results that were compatible with each outcome domain in each study were sought (e.g. for all measures, time points, analyses), and if not, the methods used to decide which results to collect.	4.5 Data extraction – Outcomes
	10b	List and define all other variables for which data were sought (e.g. participant and intervention characteristics, funding sources). Describe any assumptions made about any missing or unclear information.	4.5 Data extraction – Variables
Study risk of bias assessment	11	Specify the methods used to assess risk of bias in the included studies, including details of the tool(s) used, how many reviewers assessed each study and whether they worked independently, and if applicable, details of automation tools used in the process.	4.6 Risk-of-bias appraisal
Effect measures	12	Specify for each outcome the effect measure(s) (e.g. risk ratio, mean difference) used in the synthesis or presentation of results.	4.7 Data synthesis – Effect measures
Synthesis methods	13a	Describe the processes used to decide which studies were eligible for each synthesis (e.g. tabulating the study intervention characteristics and comparing against the planned groups for each synthesis (item #5)).	4.7 Data synthesis – Eligibility for synthesis
	13b	Describe any methods required to prepare the data for presentation or synthesis, such as handling of missing summary statistics, or data conversions.	4.7 Data synthesis – Data preparation
	13c	Describe any methods used to tabulate or visually display results of individual studies and syntheses.	4.7 Data synthesis – Tables & Figures

PRISMA 2020 Checklist

Section and Topic	Item #	Checklist item	Location where item is reported
	13d	Describe any methods used to synthesize results and provide a rationale for the choice(s). If meta-analysis was performed, describe the model(s), method(s) to identify the presence and extent of statistical heterogeneity, and software package(s) used.	4.7 Data synthesis – Narrative pooling (no meta-analysis)
	13e	Describe any methods used to explore possible causes of heterogeneity among study results (e.g. subgroup analysis, meta-regression).	4.7 / 4.8 (I^2 reported; no further exploration)
	13f	Describe any sensitivity analyses conducted to assess robustness of the synthesized results.	4.7 Sensitivity analyses
Reporting bias assessment	14	Describe any methods used to assess risk of bias due to missing results in a synthesis (arising from reporting biases).	4.6 + 4.7 (ROBINS-I reporting-bias domain)
Certainty assessment	15	Describe any methods used to assess certainty (or confidence) in the body of evidence for an outcome.	Not applicable (GRADE not performed)
**RESULTS**			
Study selection	16a	Describe the results of the search and selection process, from the number of records identified in the search to the number of studies included in the review, ideally using a flow diagram.	2. Results, opening paragraph; Figure 1
	16b	Cite studies that might appear to meet the inclusion criteria, but which were excluded, and explain why they were excluded.	Not applicable (no near-miss exclusions)
Study characteristics	17	Cite each included study and present its characteristics.	Table 1
Risk of bias in studies	18	Present assessments of risk of bias for each included study.	Supplementary Table S2
Results of individual studies	19	For all outcomes, present, for each study: (a) summary statistics for each group (where appropriate) and (b) an effect estimate and its precision (e.g. confidence/credible interval), ideally using structured tables or plots.	Not applicable (no individual comparative effect estimates)
Results of syntheses	20a	For each synthesis, briefly summarise the characteristics and risk of bias among contributing studies.	2.1 + 2.5
	20b	Present results of all statistical syntheses conducted. If meta-analysis was done, present for each the summary estimate and its precision (e.g. confidence/credible interval) and measures of statistical heterogeneity. If comparing groups, describe the direction of the effect.	Not applicable (no meta-analysis)
	20c	Present results of all investigations of possible causes of heterogeneity among study results.	Not applicable
	20d	Present results of all sensitivity analyses conducted to assess the robustness of the synthesized results.	Not applicable (aside from ROBINS-I exclusion, result unchanged)
Reporting biases	21	Present assessments of risk of bias due to missing results (arising from reporting biases) for each synthesis assessed.	Narrative in 2.5
Certainty of evidence	22	Present assessments of certainty (or confidence) in the body of evidence for each outcome assessed.	Not applicable
**DISCUSSION**			
Discussion	23a	Provide a general interpretation of the results in the context of other evidence.	3.1 What this review adds
	23b	Discuss any limitations of the evidence included in the review.	3.4 Limitations & potential biases
	23c	Discuss any limitations of the review processes used.	3.4

PRISMA 2020 Checklist

Section and Topic	Item #	Checklist item	Location where item is reported
	23d	Discuss implications of the results for practice, policy, and future research.	3.3 Clinical implications + 3.5 Future directions
**OTHER INFORMATION**			
Registration and protocol	24a	Provide registration information for the review, including register name and registration number, or state that the review was not registered.	4.1 Framework & registration (PROSPERO CRD420251082738)
	24b	Indicate where the review protocol can be accessed, or state that a protocol was not prepared.	4.1 + Supplementary Protocol S1
	24c	Describe and explain any amendments to information provided at registration or in the protocol.	Supplementary Protocol S1 "History"
Support	25	Describe sources of financial or non-financial support for the review, and the role of the funders or sponsors in the review.	Funding Statement (front matter)
Competing interests	26	Declare any competing interests of review authors.	Conflicts of Interest (front matter)
Availability of data, code and other materials	27	Report which of the following are publicly available and where they can be found: template data collection forms; data extracted from included studies; data used for all analyses; analytic code; any other materials used in the review.	Data Availability Statement (front matter)

From: Page MJ, McKenzie JE, Bossuyt PM, Boutron I, Hoffmann TC, Mulrow CD, et al. The PRISMA 2020 statement: an updated guideline for reporting systematic reviews. BMJ 2021;372:n71. doi: 10.1136/bmj.n71.
This work is licensed under CC BY 4.0. To view a copy of this license, visit https://creativecommons.org/licenses/by/4.0/
