# Supplementary material for: Vacuolated Marrow Cytopenias from Copper Deficiency to UBA1-Mutant VEXAS: Molecular Landscape, Systematic Review, and Cost-Efficient Diagnostic Algorithm
Source: Int J Mol Sci. 2025 Aug 20;26(16):8044. doi: 10.3390/ijms26168044 (PMC12386339; doi:10.3390/ijms26168044)
Supplement: Supplementary file 1 [file ijms-26-08044-s001.zip › Table S1.pdf]

Supplementary Table S1. Search strategies used for PubMed, Web of Science, and CENTRAL

Database / Interface	Search strategy (copy & paste)	Limits / Filters
PubMed	("VEXAS"[All Fields] OR "copper deficiency"[Title/Abstract] OR ((pancytopenia[Title/Abstract] OR cytopenia[Title/Abstract]) AND ("bone marrow"[Title/Abstract] AND (vacuole[Title/Abstract] OR vacuoles[Title/Abstract] OR vacuolization[Title/Abstract])))) AND ("adult"[MeSH Terms] OR "adult"[Title/Abstract]) AND ("2020/12/31"[PDAT] : "2025/05/31"[PDAT]) AND (english[Language])	Language = English<br>Publication Date = 2020-12-31 to 2025-05-31
Web of Science Core Collection	TS=(VEXAS OR "copper deficiency" OR ((pancytopenia OR cytopenia) AND "bone marrow" AND (vacuole OR vacuoles OR vacuolization OR vacuolisation))) AND TS=(adult OR adults) AND LA=(English)	Timespan = Custom range 2020-12-31 to 2025-05-31<br>Language = English
Cochrane CENTRAL	("VEXAS":ti,ab,kw OR "VEXAS syndrome":ti,ab,kw OR (copper NEXT deficiency):ti,ab,kw OR ((pancytopenia OR cytopenia OR cytopenias) AND ("bone marrow" OR marrow) AND vacuol*):ti,ab,kw)	Language = English; Publication Year = 2020-12-31 to 2025-05-31<br>Population = Adult
