# Supplementary material for: Vacuolated Marrow Cytopenias from Copper Deficiency to UBA1-Mutant VEXAS: Molecular Landscape, Systematic Review, and Cost-Efficient Diagnostic Algorithm
Source: Int J Mol Sci. 2025 Aug 20;26(16):8044. doi: 10.3390/ijms26168044 (PMC12386339; doi:10.3390/ijms26168044)
Supplement: Supplementary file 1 [file ijms-26-08044-s001.zip › Table S2.pdf]

Supplementary Table S2. Risk-of-bias summary for the 24 included studies

Each row lists First Author, Year, and Disease cohort. Colors represent reviewer judgments across the seven ROBINS-I domains.

Study	Confounding	Selection of participants	Classification of interventions / exposures	Deviations from intended	Missing data	Measurement of outcomes	Selection of reported results
1 Gurnari 2021	Low	Low	Low	Low	Moderate	Moderate	Low
2 Uchino 2021	Low	Low	Low	Low	Low	Moderate	Low
3 Halfdanarson 2009	Low	Low	Low	Low	Moderate	Moderate	Low
4 Halfdanarson 2008	Low	Low	Low	Low	Moderate	Moderate	Low
5 Huff 2007	Low	Low	Low	Low	Moderate	Moderate	Low
6 Gurnari 2021	Low	Low	Low	Low	Moderate	Moderate	Low
7 Vitale 2025	Low	Low	Low	Low	Moderate	Moderate	Low
8 Johansen 2025	Low	Low	Low	Low	Moderate	Serious	Low
9 Hadjadj 2024	Low	Low	Low	Low	Moderate	Moderate	Low
10 Maeda 2024	Low	Low	Low	Low	Moderate	Moderate	Low
11 Kusne 2024	Low	Low	Low	Low	Serious	Moderate	Low
12 Wolff 2024	Low	Low	Low	Low	Moderate	Moderate	Low
13 Beck 2023	Low	Low	Low	Low	Low	Moderate	Low
14 Mascaro 2023	Low	Low	Low	Low	Moderate	Moderate	Low
15 Hines 2023	Low	Low	Low	Low	Moderate	Moderate	Low
16 Islam 2022	Low	Low	Low	Low	Moderate	Moderate	Low
17 Mekinian 2022	Low	Low	Low	Low	Moderate	Moderate	Low
18 Georgin-Lavialle 2022	Low	Low	Low	Low	Moderate	Moderate	Low

19 Comont 2022	Low	Low	Low	Low	Moderate	Moderate	Low
20 Ferrada 2022	Low	Low	Low	Low	Serious	Moderate	Low
21 Tsuchida 2021	Low	Low	Low	Low	Moderate	Moderate	Low
22 Ferrada 2021	Low	Low	Low	Low	Moderate	Moderate	Low
23 Gurnari 2021	Low	Low	Low	Low	Moderate	Moderate	Low
24 Beck 2020	Low	Low	Low	Low	Moderate	Moderate	Low
